# Laccases as palladium oxidases†
†Electronic supplementary information (ESI) available: Experimental procedures, synthesis of catalysts molecules, enzyme activity assay, bleaching experiments, oxygraph traces, oxidation of veratryl alcohol assay, inhibition experiments, electrophoresis. See DOI: 10.1039/c4sc02564d



**DOI:** 10.1039/c4sc02564d

**Published:** 2014-11-14

**Authors:** Yasmina Mekmouche, Ludovic Schneider, Pierre Rousselot-Pailley, Bruno Faure, A. Jalila Simaan, Constance Bochot, Marius Réglier, Thierry Tron

**Affiliations:** a Aix Marseille Université , CNRS , Centrale Marseille , ISM2 UMR 7313 , 13397 , Marseille , France . Email: y.mekmouche@univ-amu.fr ; Email: thierry.tron@univ-amu.fr

## Abstract

The first example of a coupled catalytic system involving an enzyme and a palladium(ii) catalyst competent for the aerobic oxidation of alcohol in mild conditions is described. In the absence of dioxygen, the fungal laccase LAC3 is reduced by a palladium(0) species as evidenced by the UV/VIS and ESR spectra of the enzyme. During the oxidation of veratryl alcohol performed in water, at room temperature and atmospheric pressure, LAC3 regenerates the palladium catalyst, is reduced and catalyzes the four-electron reduction of dioxygen into water with no loss of enzyme activity. The association of a laccase with a water-soluble palladium complex results in a 7-fold increase in the catalytic efficiency of the complex. This is the first step in the design of a family of renewable palladium catalysts for aerobic oxidation.

## Abstract 2

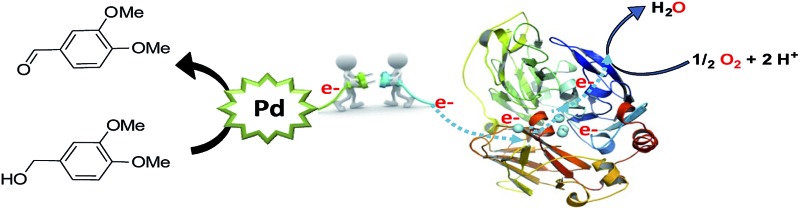
Combining a palladium-based complex with a laccase allows the oxidation of an alcohol substrate at room temperature and atmospheric pressure.

## Introduction

There is a constant need for the development of new robust catalysts for the selective oxidation of organic compounds for the production of pharmaceuticals, agrochemicals and fine chemicals.^1^ Recently, substantial advances have occurred in Pd-catalyzed oxidative reactions.^2^ Particularly, the Pd-catalyzed aerobic oxidation of a wide range of alcohols is now possible with O_2_ as the oxidant in organic solvents^2*g*^ as well as in water.^2*h*^ However, although they ensure high conversion yields, experimental conditions such as the use of organic solvents, strongly basic pH and elevated pressure and/or temperature are not compatible with the increasing demand of society for more sustainable chemistry.^1^ Therefore, it is quite topical to find systems which efficiently couple the catalyst to O_2_ while functioning in water, at a moderate pH, ambient temperature and atmospheric pressure.^3^ Over the past few years, methodologies combining both homogeneous and enzymatic catalysis have been developed in order to exceed the limitations encountered separately in each field. Appearing as highly promising sustainable alternatives to traditional catalysts, artificial enzymes often combine a transition metal catalyst moiety to a protein environment.^4^ Laccases are biocatalysts that use molecular oxygen to oxidize a wide range of substrates, including metal ions, producing water as the co-product of substrate oxidation.^5^ Their substrate oxidation capabilities can be largely enhanced by combining them with redox mediators.^6^ We report here on an unprecedented bimolecular system made of a redox enzyme and a water soluble palladium complex that oxidizes an alcohol into the corresponding aldehyde at ambient temperature and atmospheric pressure.

## Results and discussion

**Scheme 1:**
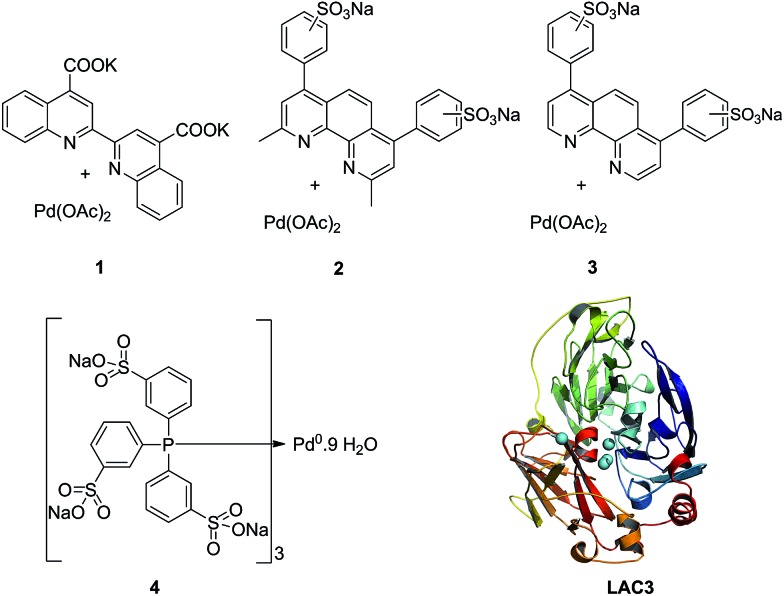
Water soluble palladium complexes (**1–4**) and model of the structure of LAC3. The copper ions are depicted as spheres in cyan.

The enzyme LAC3 is a typical fungal laccase expressed heterologously and purified with high yield (Scheme 1).^7,8^ LAC3 works under acidic conditions and is stable for days at moderate temperatures (*e.g.* 40 °C).^9^

**Scheme 2:**
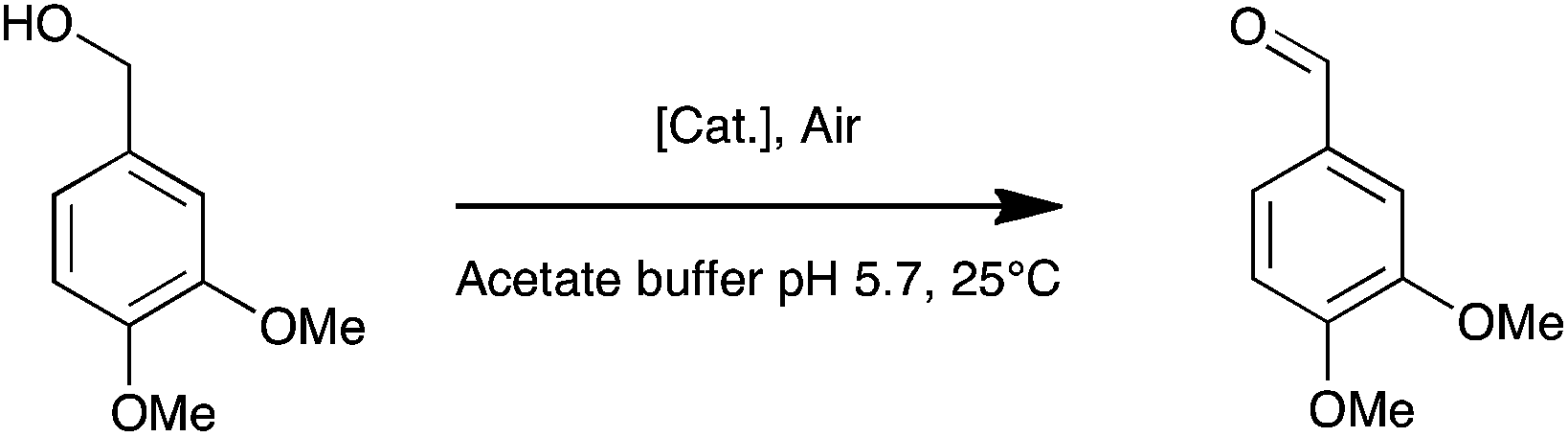
Oxidation of veratryl alcohol.

It contains a surface located type 1 copper (T1, substrate oxidation center) connected to a trinuclear center (dioxygen reduction center) structured between a type 3 pair of anti-ferromagnetically coupled copper ions (T3) and a type 2 (T2) copper ion.^5^ Complexes **1**, **2**, and **3** are palladium(ii) water soluble complexes containing respectively a 2,2′-biquinoline-4,4′-dicarboxylate ligand, a bathocuproin disulfonate ligand or a bathophenanthroline disulfonate ligand; complex **4** is a palladium(0) complex containing a triphenyl phosphine sulfonated ligand (Scheme 1, see ESI† for details). All of these complexes are known to oxidize primary and secondary alcohols under rather harsh conditions (*i.e.* high temperature and high pressure).^2h,10–12^ The model reaction targeted in this study is the oxidation of veratryl alcohol – a model compound of lignin – into veratryl aldehyde (Scheme 2). Laccases, as verified for LAC3, are unable to oxidize this substrate.

**Fig. 1:**
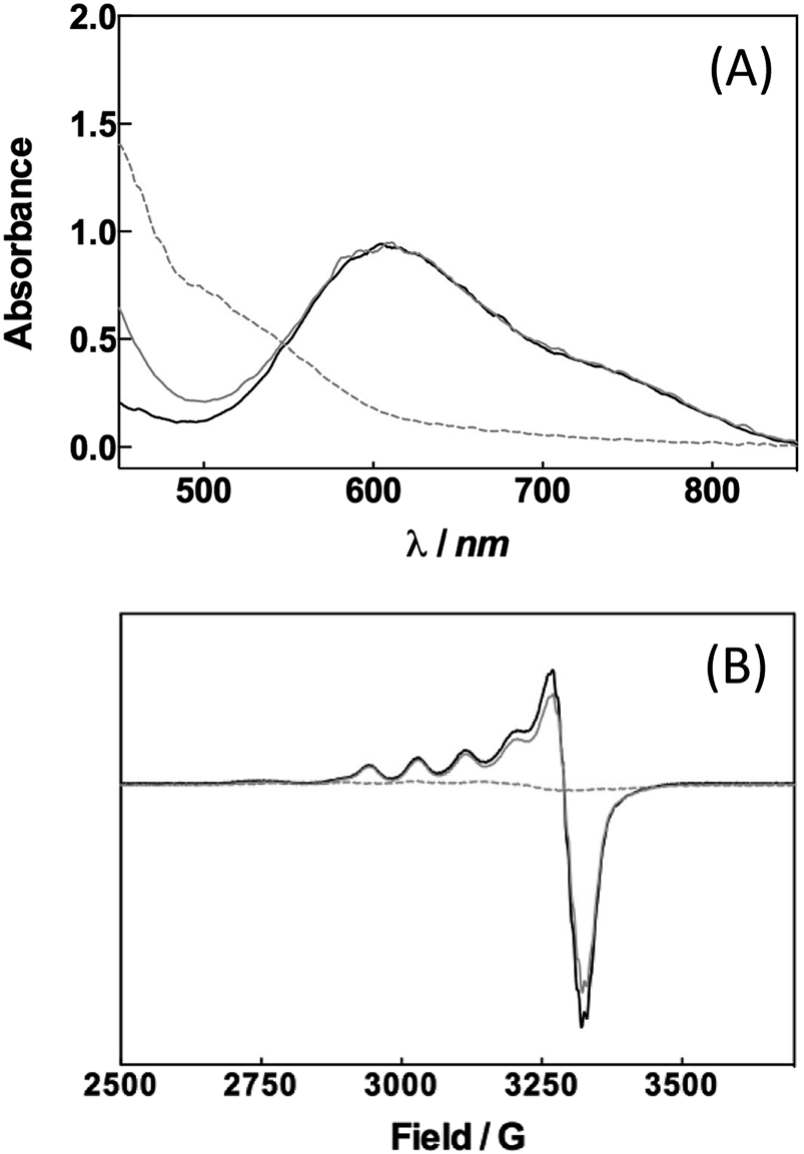
Evolution of the spectroscopic features of LAC3 in the presence of complex **1** and substrate. A. UV/VIS spectra. B. X-band ESR spectra of frozen solutions. Temperature 115 K, microwave power 20 mW, modulation 3 G, gain 10^5^. [LAC3] = 209 μM in acetate buffer 33 mM pH 5.7 (solid black) incubated anaerobically with 10 equivalents of complex **1** (solid grey) and in the presence of 200 mM of veratryl alcohol (dotted grey).

**Scheme 3:**
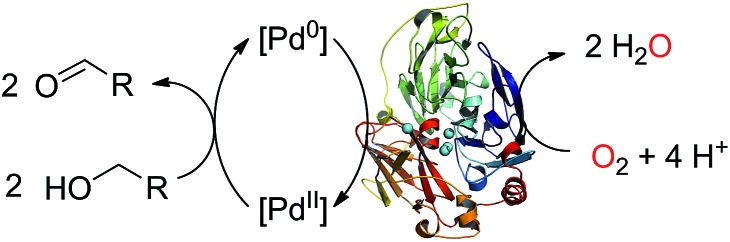
Aerobic oxidation of alcohol by a Pd/laccase hybrid system.

Can a laccase be reduced by a palladium species? Exploiting the spectroscopic properties of the Cu(ii) sites present in the enzyme we first monitored changes in the redox state of LAC3 linked to the presence of different palladium species. Under anaerobic conditions and upon addition of 10 equivalents of freshly prepared solutions of the biquinoline ligand, complex **1** or veratryl alcohol to LAC3, no change in the in the UV-visible spectrum (transition at 610 nm, *ε* = 5600 M^–1^ cm^–1^, characteristic of the Cu(ii) T1 copper, Fig. 1A) nor in the Electron Spin Resonance (ESR) spectrum (Fig. 1B) were observed. On the other hand, adding both **1** and an excess of substrate to a LAC3 solution resulted in a complete bleaching of the 610 nm band as well as in the total disappearance of both the Cu(ii) T1 and T2 ESR signatures (Fig. 1). In other words, in the absence of a final electron acceptor (O_2_) the enzyme gets reduced only in the presence of both **1** and substrate. As none of the individual components is the reductant this suggests that the reductant is a product of the reaction between **1** and the substrate, probably a reduced Pd species. Strongly supporting this hypothesis, it is worth noting that replacing “**1** + substrate” by the water soluble palladium(0) complex **4** resulted similarly in the full reduction of LAC3 (Fig. SI1†). Upon re-oxygenation of the samples containing either complex **1** or complex **4**, all the spectroscopic features reappeared as well as the blue color of the enzyme attesting for the re-oxidation of the laccase.^13–15^ All together, these results support the formation of a transient Pd(0) intermediate during the oxidation of veratryl alcohol by complex **1** and the re-oxidation of Pd(0) by the laccase followed by the catalytic reduction of dioxygen into water (Scheme 3).

**Fig. 2:**
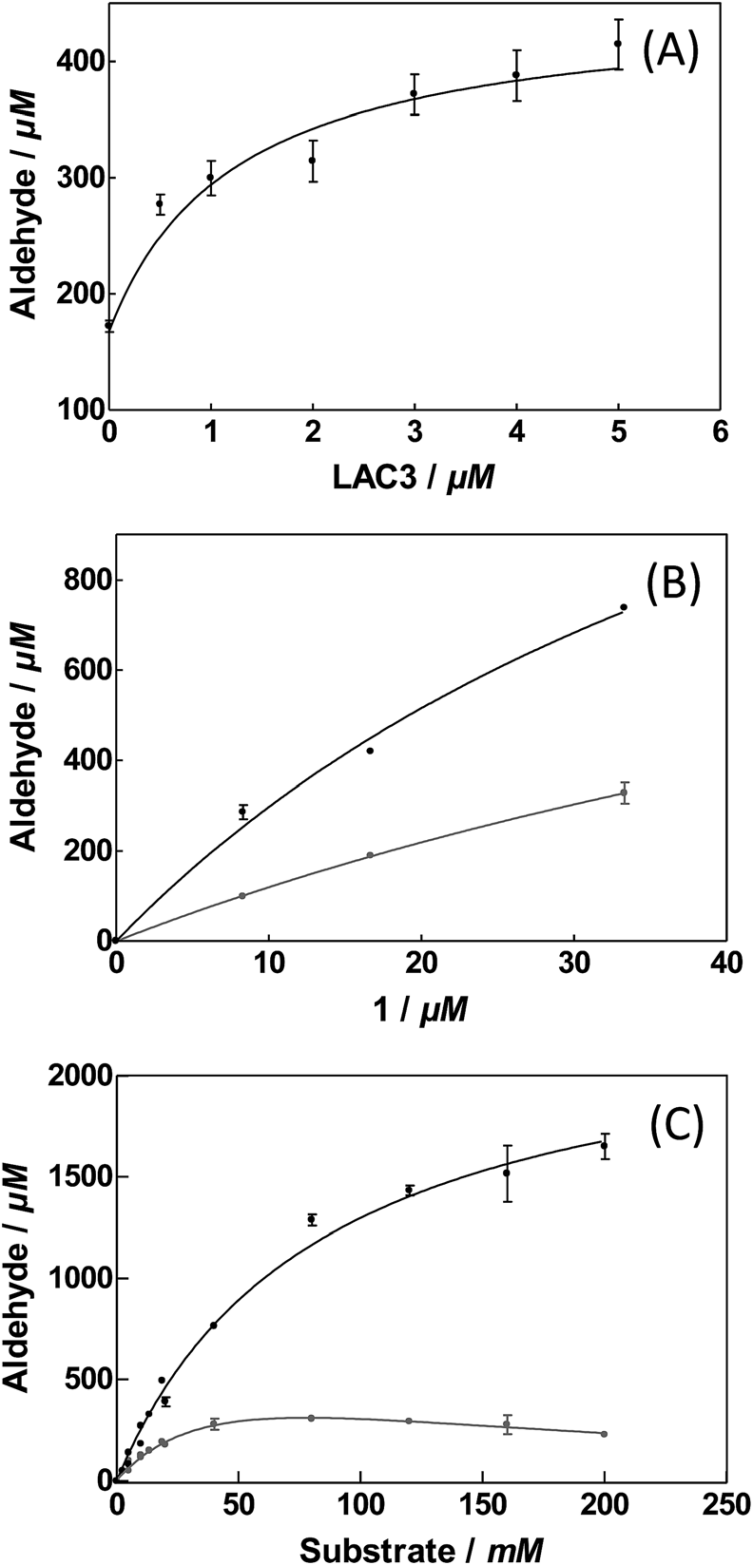
Veratryl alcohol oxidation in acetate buffer 33 mM pH 5.7 at 25 °C for 24 hours. (A) As a function of LAC3. [LAC3] = 0 to 5 μM and [**1**] = 50 μM; [substrate] = 20 mM. (B) As a function of **1**. [LAC3] = 5 μM and [**1**] = 0 to 100 μM; [substrate] = 20 mM. (C) As function of substrate. [LAC3] = 5 μM and [**1**] = 50 μM [substrate] = 0 to 200 mM. Grey circles: complex **1** alone; black circles: complex **1** in the presence of LAC3. Product formation was determined by HPLC (Fig. SI3†). Values presented are an average of three sets of independent reactions.

To evaluate the influence of LAC3 on the oxidation of veratryl alcohol by a palladium complex we explored the catalytic properties of complex **1** alone and combined with LAC3 under conditions where each of the partners (LAC3 and **1**) retains some activity (*e.g.*, moderate acidic conditions, atmospheric pressure, ambient *T* °C). Using a Clark electrode we first measured the variation of dioxygen concentration in an air-saturated complex **1** (50 μM) buffered solution (33 mM acetate set at pH 5.7 at 25 °C) upon addition of veratryl alcohol (50 mM) and LAC3 (5 μM). In the absence of both laccase and substrate no dioxygen was consumed. In the presence of substrate the concentration of dissolved dioxygen decreased attesting a probable re-oxidation of **1** along with substrate oxidation.^2^ Indeed, the two stages of the catalytic mechanism of palladium catalysts are (i) oxidation of an organic molecule by the metal center and (ii) oxidation of the reduced catalyst by an oxidant.^2^ Upon addition of LAC3 to the mixture the rate of dioxygen consumption increased substantially (Fig. SI2†). A subsequent addition of sodium azide, a potent inhibitor of laccase, led to a net decrease in dioxygen consumption attesting for the active participation of LAC3 in the process (Fig. SI2†). With this catalytic hybrid system (*i.e.* substrate + **1** + LAC3 + O_2_) veratryl aldehyde was found to accumulate in a time dependent manner (Fig. 2). At room temperature, atmospheric pressure and in buffered water, complex **1** (2.5‰) oxidizes veratryl alcohol into the corresponding aldehyde (172 ± 0.11 μM, 3.4 TON, 0.14 ± 0.01 h^–1^) with low efficiency.‡
‡As compared to data previously published for complex **1** (ref. 10) or other water soluble palladium-based catalysts^2h,12^ operated in much harsher conditions, *e.g.* cat. load 1%, pH 11, 41 bars of air and at 125 °C. Incubating complex **1** with increasing amounts of LAC3 (0 to 5 μM) resulted in the increased production of veratryl aldehyde reaching up to 415 ± 53 μM in 24 hours (8.3 TON, 0.35 ± 0.04 h^–1^) in the presence of 5 μM LAC3 (Fig. 2 A). Since LAC3 has no detectable activity towards veratryl alcohol or veratryl aldehyde, the observed immediate improvement of the catalytic efficiency was attributed to an improvement in the activity of complex **1**. A decrease in the formation of veratryl aldehyde observed in the presence of NaN_3_ (Fig. SI3†) confirmed the active participation of LAC3 in the oxidation process (*i.e.* as a redox enzyme and not only as a protein scaffold). Beyond [LAC3] = 5 μM, a decrease in the activity of **1** was observed (Fig. SI4†). Several examples of palladium complexes interacting with peptides and proteins through cysteine and histidine residues have been reported in the literature^16^ including palladium anticancer complexes with HSA^17^ and a structured Pd(allyl)–ferritin complex.^18^ As labile metal coordination ligands are necessary for the exogenous substrate to bind with the complex,^2b,2d,12a,19^ catalysis can be prevented in the case of an exchange with more stable protein ligands. Therefore, the decrease of activity we observed could be consecutive to an inactivation of the catalyst by the enzyme rather than to an enzyme inactivation. Note that, after the catalysis experiments had been carried out, no change in the electrophoretic profile (Fig. SI5†) nor any change in the phenol-oxidase activity of LAC3 were observed suggesting that known palladium salts-induced modifications of proteins such as proteolysis^20^ or oligomerisation^21^ did not occur significantly here.

Increasing the ratio of **1** *vs.* LAC3 from 10 to 20 yielded more products without any improvement of the catalytic efficiency (Fig. 2B). Maintaining this ratio to a fixed value (**1**/LAC3 = 10) with a [**1**] ranging from 25 μM to 100 μM did not result in a drastic change in the catalytic properties of the bimolecular system (Table SI1†). Similarly, extending the catalysis up to 72 hours yielded more products while retaining 70% of the catalytic efficiency (Fig. SI6†). Varying the substrate concentration we found that the activity of complex **1** inhibited above 50 mM of veratryl alcohol whereas this inhibition was prevented in the presence of LAC3 (Fig. 2C). For 200 mM of substrate, aldehyde production increased from 226 ± 16 μM (0.20 ± 0.01 h^–1^) in the absence of enzyme to 1650 ± 110 μM (1.4 ± 0.1 h^–1^) in the presence of enzyme. All together, the combination of LAC3 with **1** accounts for up to a 7-fold improvement in the catalytic efficiency of the palladium complex alone. For the sake of comparison, we checked the activity of **1** in the presence of an equimolar amount of benzoquinone (a well known Pd(0) reoxidation agent). Under our experimental conditions benzoquinone has no effect on the Pd complex activity (Fig. SI7†).

Variations of the ligand in the palladium complex were found to affect the catalytic activity of the Pd/enzyme hybrid. For 100 mM of substrate, a modest improvement was obtained with complex **2** (the basal activity of which is twice that observed with complex **1**), reaching a TON of 33 and a TOF of 1.4 h^–1^ once combined with LAC3 (Table 1). Under the same conditions, complex **3** did not yield any product. These results fit the recent proposition of Arends *et al.* ^11^ who suggested that substituents at the α-position of the pyridine ring, here a phenyl or methyl group respectively present in complexes **1** and **2** and absent in complex **3**, are of importance for the activity and the stability of the catalyst.

**Table 1:** Aerobic veratryl alcohol oxidation in 33 mM acetate buffer pH 5.7, at room temperature and atmospheric pressure

Complex^*a*^	TON^*b*^		TOF^*c*^ (h^–1^)	
	–LAC3	+LAC3	–LAC3	+LAC3
**1**	5 ± 1	24 ± 2	0.2 ± 0.04	1.0 ± 0.1
**2**	10 ± 1	33 ± 3	0.4 ± 0.04	1.4 ± 0.1
**3**	No product	No product	No product	No product

^*a*^[LAC3] = 5 μM or/and [catalyst] = 50 μM incubated with 100 mM veratryl alcohol for 24 hours at 25 °C with gentle shaking.

^*b*^TON = turn over number = moles of aldehyde/moles of palladium.

^*c*^TOF = turn over frequency = turn over number/time of catalysis. Values presented here are an average of three sets of independent reactions.

Interestingly, aldehydes can be quantitatively produced from alcohols *via* metal-free oxidation with, for example, a TEMPO/HNO_3_/HCl catalytic system in acetonitrile^22^ or with a TEMPO/laccase catalytic system in buffered water.^23^ The yield of aldehyde achieved with a Pd complex/laccase hybrid system is low (due in part to the modest catalyst load used here). However, its alcohol oxidase activity (33 TON, 1.4 h^–1^) can be compared with those previously reported for similar palladium complexes (97 TON, 5.4 h^–1^)^10*b*^ or for a nanostructure made of a thermo resistant ferritin module containing a Pd salt (138 TON, 5.8 h^–1^).^18^ This comparison appears of particular significance if one considers that, in our experiments, no extra energy in the form of heating or an increase in pressure was applied to the system. Eventually, compared to TEMPO based systems, considering the richness of Pd-centered chemistry the improvement of Pd/laccase hybrid systems through chemogenetic evolution schemes appear worthy of consideration.

## Conclusions

In summary, we have described here for the first time the combination of water soluble palladium(ii) complexes to an oxidoreductase resulting in the promotion (up to 7-fold) of the aerobic oxidation of alcohol at room temperature and ambient pressure with no need for a harmful oxidant. In this hybrid system, the substrate is oxidized by a palladium complex probably cycling between the Pd(ii)/Pd(0) redox states assisted, *via* the enzyme, by a four-electron reduction of dioxygen into water. Therefore, dioxygen is used as a renewable, cheap and clean oxidant in particularly eco-friendly conditions. The enzyme and the palladium catalysts form bimolecular systems operating without any extra mediator and without loss of the enzyme integrity. Beyond the work of Bäckvall *et al.* who used a biomimetic coupled catalytic system involving a palladium catalyst, a hydroquinone as a mediator and a cobalt porphyrin as the oxygen activating agent,^24^ the work presented here is the first example of a native oxidoreductase promoting the regeneration of a palladium catalyst. From a biotechnological point of view, the palladium catalyst can be compared to a redox mediator cycling between laccase and the lignin model compound veratryl alcohol,^5b,6^ with the advantage of selective metal-controlled chemistry. Pd/laccase hybrids appear thus as potential valuable chemo-enzymatic tools for the synthesis of fine chemicals. Improvements of the current bimolecular system are under investigation. One such improvement currently being studied in our laboratory involves grafting of the enzyme surface with the catalyst.

## References

[cit1] Arends I. W. C. E. and Sheldon R. A., in Modern oxidation methods, ed. J. E. Bäckvall, Wiley-VCH, Weinheim, 2004, vol. 1, pp. 83–118.

[cit2] Heumann A., Jens K.-J., Réglier M. (1994). Prog. Inorg. Chem..

[cit3] Parmeggiani C., Cardona F. (2012). Green Chem..

[cit4] Letondor C., Ward T. R. (2006). ChemBioChem.

[cit5] Solomon E. I., Sundaram U. M., Machonkin T. E. (1996). Chem. Rev..

[cit6] Cañas A. I., Camarero S. (2010). Biotechnol. Adv..

[cit7] Klonowska A., Gaudin C., Asso M., Fournel A., Réglier M., Tron T. (2005). Enzyme Microb. Technol..

[cit8] Mekmouche Y., Zhou S., Cusano A. M., Record E., Lomascolo A., Robert V., Simaan A. J., Rousselot-Pailley P., Chaspoul F., Tron T. (2014). J. Biosci. Bioeng..

[cit9] Cusano A. M., Mekmouche Y., Meglecz E., Tron T. (2009). FEBS J..

[cit10] Buffin B. P., Clarkson J. P., Belitz N. L., Kundu A. (2005). J. Mol. Catal. A: Chem..

[cit11] Arends I. W. C. E., ten Brink G.-J., Sheldon R. A. (2006). J. Mol. Catal. A: Chem..

[cit12] Sheldon R. A., Arends I. W. C. E., ten brink G.-J., Dijksman A. (2002). Acc. Chem. Res..

[cit13] Simaan A. J., Mekmouche Y., Herrero C., Moreno P., Aukauloo A., Delaire J. A., Réglier M., Tron T. (2011). Chem.–Eur. J..

[cit14] Blackburn N. J., Ralle M., Hassett R., Kosman D. J. (2000). Biochemistry.

[cit15] Hakulinen N., Kiiskinen L. L., Kruus K., Saloheimo M., Paananen A., Koivula A., Rouvinen J. (2002). Nat. Struct. Biol..

[cit16] Wang Y., Feng L., Zhang B., Wang X., Huang C., Li Y., Du W. (2011). Inorg. Chem..

[cit17] Divsalar A., Bagheri M. J., Saboury A. A., Mansoori-Torshizi H., Amani M. J. (2009). J. Phys. Chem. B.

[cit18] Kanbak-Aksu S., Nahid Hasan M., Hagen W. R., Hollmann F., Sordi D., Sheldon R. A., Arends I. W. C. E. (2012). Chem. Commun..

[cit19] Popp B. V., Stahl S. S. (2009). Chem.–Eur. J..

[cit20] Rajković S., Živković M. D., Kállay C., Sóvágó I., Djuran M. I. (2009). Dalton Trans..

[cit21] Zang Q., Zhong W., Xing B., Tang W., Chen Y. (1998). J. Inorg. Biochem..

[cit22] Rahimi A., Kim A. H., Ralph J., Stahl S. S. (2013). J. Am. Chem. Soc..

[cit23] Fabbrini M., Galli C., Gentili P., Macchitella D. (2001). Tetrahedron Lett..

[cit24] Verboom R. C., Slagt V. F., Bäckwall J.-E. (2005). Chem. Commun..

