# Supplementary material for: Laccases as palladium oxidases
Source: Chem Sci. 2014 Nov 14;6(2):1247–51. doi: 10.1039/c4sc02564d (PMC5811087; doi:10.1039/c4sc02564d)
Supplement: Supplementary file 1 [file SC-006-C4SC02564D-s001.pdf]

Electronic Supplementary Material (ESI) for Chemical Science.
This journal is © The Royal Society of Chemistry 2014

Laccases as palladium oxidase

Yasmina Mekmouche*, Ludovic Schneider, Pierre Rousselot-Pailley, Bruno Faure, A. Jalila Simaan, Constance Bochot, Marius Réglier and Thierry Tron*

Supporting information

Synthetic procedures

Complexes **1**[1], **2**[2] and **3**[3] were synthetized according to adapted published protocols. Tris(3,3′,3″-phosphinidynetris(benzenesulfonato)palladium(0) nonasodium salt nonahydrate] (complex **4**), bathophenanthrolinedisulfonic acid disodium salt , bathocuproin disulfonic acid disodium, 2,2′-Biquinoline-4,4′-dicarboxylic acid dipotassium salt, triphenylphosphine-3,3′,3′′-trisulfonic acid trisodium salt ligand, veratryl alcohol, veratryl aldehyde and all chemicals were purchased from Sigma Aldrich and used without further purification.
Pd(II) acetate and 1.1 equivalent of ligand were placed in an agate mortar with 2-3 drops of water and finely ground. 3 drops of NaOH 1 M were added to the resulting paste, transferred to a metal-free propylene tube and diluted to 25 mL with water and shaken at 28 °C on a wrist-action shaker for 24 hours. The resulting mixture was then filtered through a nylon membrane (0.2 µm porosity), precipitated with acetone, dried with diethyl ether and used for catalysis.
Laccase LAC3 was heterologously expressed in *Aspergillus niger* and purified according to previously published procedures[4].

Standard enzyme activity assay.

Laccase activity was assayed at 30°C using syringaldazine (SGZ) as substrate. Oxidation of SGZ was detected by following the absorbance at 525 nm ($\varepsilon_{525\ nm}$ = $6.5x10^4$ $M^{-1}.cm^{-1}$) during 2 min using a spectrophotometer (Carry 50, Varian). The reaction mixture (1 mL) contained 10 µL of appropriately diluted enzyme sample, 960 µL of acetate buffer (100 mM pH 5.7) and 30 µL of 0.8 mg/mL SGZ in MeOH at 30°C. The SGZ was added to initiate the reaction. One unit (U) of laccase was defined as one micromole of substrate oxidized per minute in these described conditions.

Bleaching experiments

UV/VIS. Experiments were performed on a VARIAN Cary 50 spectrophotometer. With complex **1**: 209 µM laccase LAC3, 2 mM complex **1** in the absence or presence of 200 mM veratryl alcohol in acetate buffer (33 mM pH 5.7) at 25 °C. With complex **4**: 70 µM laccase and 0 to 700 µM of complex **4** in acetate buffer (33 mM pH 5.7) at 25 °C under anaerobic conditions (Fig. SI1A). Kinetics of reduction were followed at 610 nm.
ESR. Spectra were obtained using a BRUKER EMX9/2.7 spectrometer equipped with a B-VT2000 digital temperature controller (100-400 K). With complex **1**: 209 µM LAC3, 10 equivalents of complex **1** in the absence or presence of veratryl alcohol 200 mM in 33 mM acetate buffer pH 5.7. With complex **4**: 180 µM of laccase, 10 equivalents of complex **4** in 33 mM acetate buffer pH 5.7. Samples were placed in J. Young valved ESR tubes under inert atmosphere and analysed as frozen solutions (115 K, Fig. SI1B).

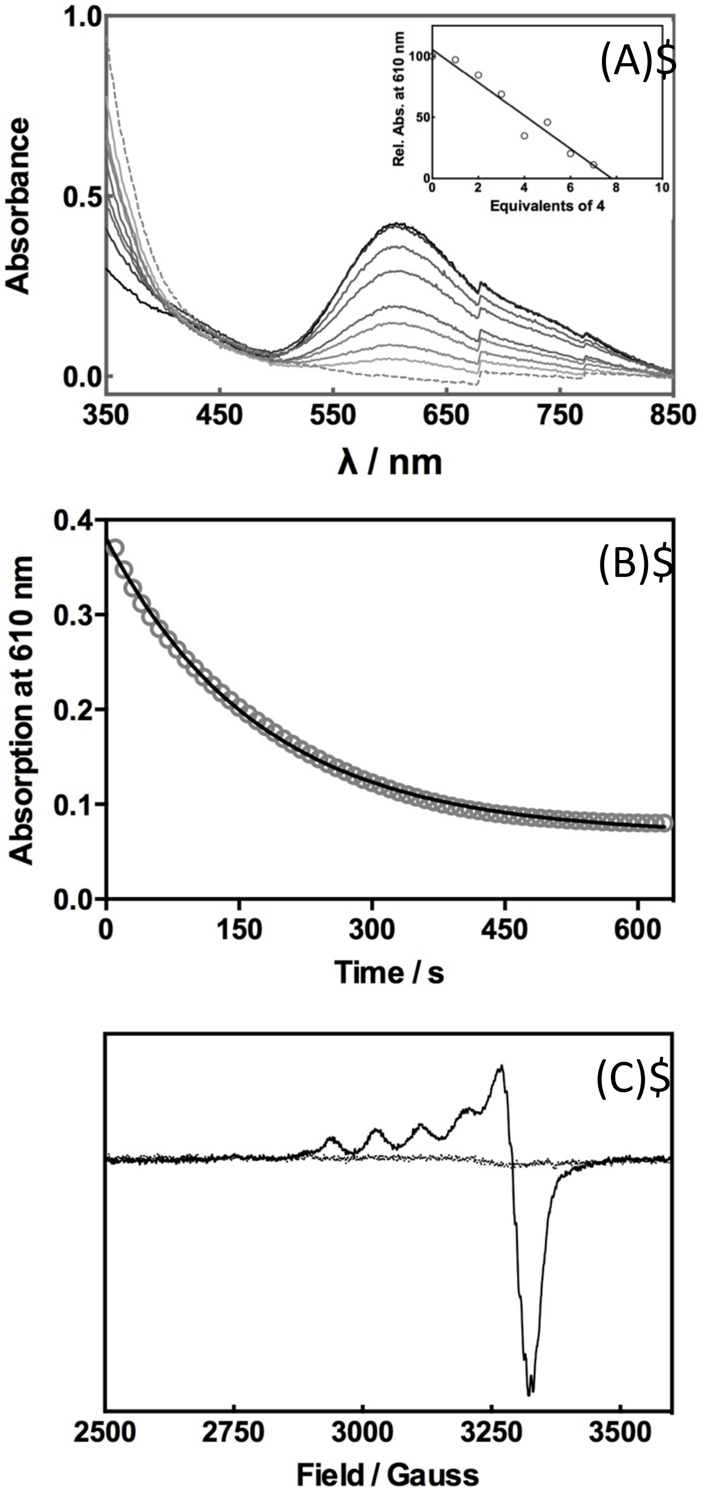

Figure SI1. Reduction of LAC3 by complex **4**. A. Bleaching of the absorption band characteristic of the T1 site Cu(II) of laccase by complex **4**; inset: decrease in absorption (%) as function of the number of equivalent of complex **4**. B. Transient kinetics obtained after mixing degassed solutions of oxidized laccase (70 μM) with 10 equivalents of complex **4** in 33 mM acetate buffer pH 5.7 at 25°C. Absorption changes monitored at 610 nm; a half-life time of 120 ± 7 sec was estimated for the bleaching of the T1 site. C. X-band ESR spectra recorded from frozen solutions; (—) before and (---) after incubation with 10 equivalents of complex **4**. Temperature 115 K, microwave power 20 mW, modulation 3 G, gain 10^5. No reduction occurs in the presence of triphenylphosphine-3,3′,3′′-trisulfonic acid trisodium salt ligand.

Dioxygen consumption experiments.

Dioxygen consumption was measured by potentiometry using a model 781 oxygen meter (Strathkelvin Instruments) with a micro Clark electrode fitted to a temperature controlled glass chamber (1.0 ml). A solution of 50 µM complex **1**, 50 mM veratryl adehyde was placed in the chamber. A decrease in dioxygen concentration was followed (250µM initial concentration at 25 °C) then laccase (5 µM) was added to the mixture followed by NaN_3 (100 µM).

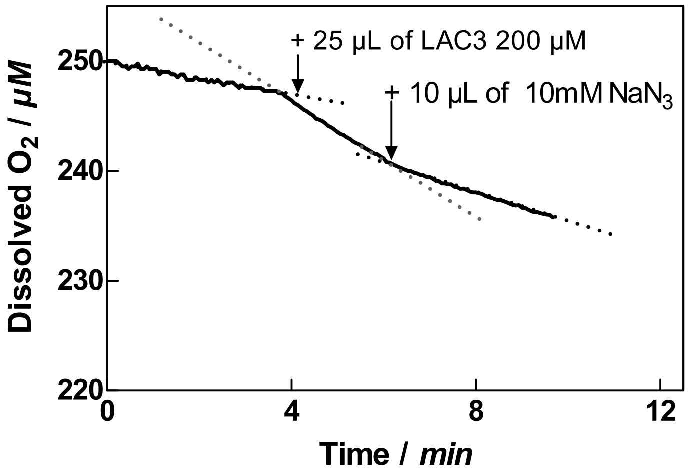

Figure SI2. Influence of LAC3 activity on the dioxygen consumption rate during veratryl alcohol oxidation by complex **1**. Rates were measured with a Clark type electrode in a temperature controlled glass vessel (T=25°C) containing 50 µM complex **1** with 50 mM veratryl alcohol in 1.0 ml of 33 mM acetate buffer pH 5.7.

Oxidation of veratryl alcohol assay.

In a typical experiment, oxidation of veratryl alcohol was performed in 15 mL tube at 25 °C with gentle constant shaking. Freshly prepared complex solution (0 to 100 µM based on palladium) was incubated with veratryl alcohol (0 to 200 mM) in 500 µL 33 mM acetate buffer pH 5.7 for 24 hours. When laccase LAC3 was present, 0 to 5 µM (based on T1 extinction coefficient ε_{610nm} = 5600 $M^{-1}.cm^{-1}$) were added to the mixture. Samples were then applied onto a C18 nucleosil column (300 A, 250*4.6 mm) after extraction with an ethylacetate solution containing 1 mM benzophenone as a standard for quantification, samples were analysed by High Performance Liquid Chromatography (Waters 2695 equipped with a photodiode array detector). The aldehyde concentration was determined spectrophotometrically at 310 nm from a standard curve performed with commercial aldehyde solutions.

Influence of LAC3 and NaN_3 on product formation.

Oxidation of veratryl alcohol was performed in 2 mL 1 cm path-lengh UV-Visible cell with gentle constant shaking on a Varian Cary 50 bio equipped with a thermostat and followed at 310 nm (Veratryl aldehyde : λ 310 nm = 9300 $M^{-1}.cm^{-1}$). In a typical experiment, 50 µM complex **1** with 20 mM substrate in presence of LAC3 (0 to 10 µM) in 2 mL of 33 mM acetate buffer pH 5.7 at 25 °C. For inhibition studies, 100 µM NaN_3 were added to the mixture. *Vi* (initial velocities) were extracted with a linear fitting of the curve obtained after 150 min of reaction.

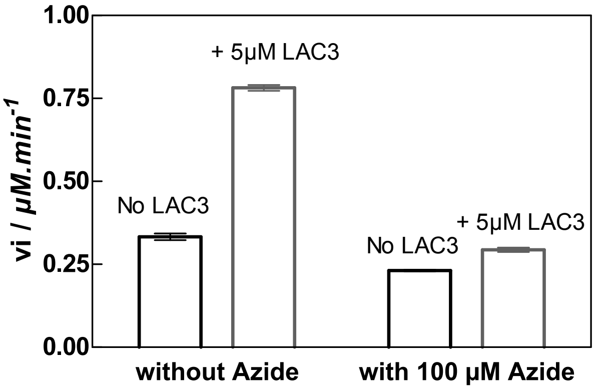

Figure SI3. Influence of NaN_3 on veratryl aldehyde production. Rates were measured by UV-Vis spectroscopy in a 2 mL (1 cm-path length) quartz cuvette containing 50 µM complex **1** and 50 mM veratryl alcohol in 33 mM acetate buffer pH 5.7.

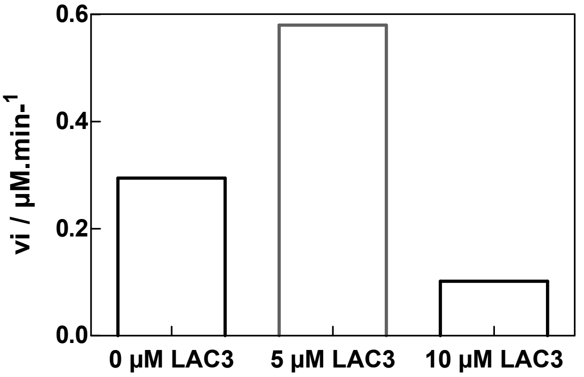

Figure SI4. Influence of LAC3 concentration on product formation. Rates were measured by UV-Vis spectroscopy in a 2 mL (1 cm-path length) quartz cuvette containing 50 µM complex 1 and 20 mM veratryl alcohol in 33 mM acetate buffer pH 5.7.

Electrophoresis experiments

Standard sodium dodecyl sulfate polyacrylamide gel electrophoresis (SDS-PAGE) (7.5 % acrylamide) were used for the separation of denatured protein samples (10 min at 100°C in Laemmli loading buffer)[5]. 20 µL of each sample were loaded on gel after the thermal denaturation under reducing conditions. The gels were then stained with coomassie and decolorized with a EtOH/Acetic acid/ H_2O (4/1/5) solution.

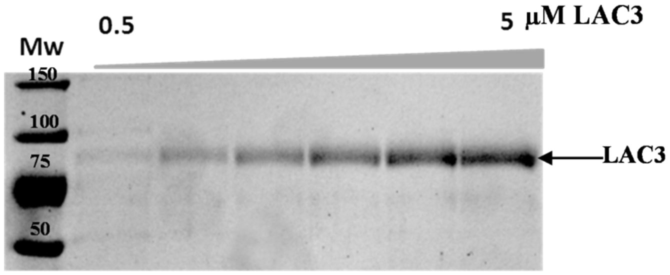

Figure SI5. Electropheric profile of LAC3 (0.5; 1; 2; 3; 4; 5 µM) in the presence of 50 µM complex **1** and 20 mM substrate after 72 hours of reaction at 25°C in 33 mM acetate buffer pH5.7. Mw = molecular weight in kDa.

Influence of the reaction time on catalytic efficiencies

Oxidation of veratryl alcohol was performed in 15 mL tube at 25 °C with gentle constant shaking. 50 µM of freshly prepared complex solution was incubated with 20 mM veratryl alcohol in 500 µL 33 mM acetate buffer pH 5.7 for 24, 44 and 72 hours . When laccase LAC3 was present, 0 to 5 µM were added to the mixture.

A. B. C.

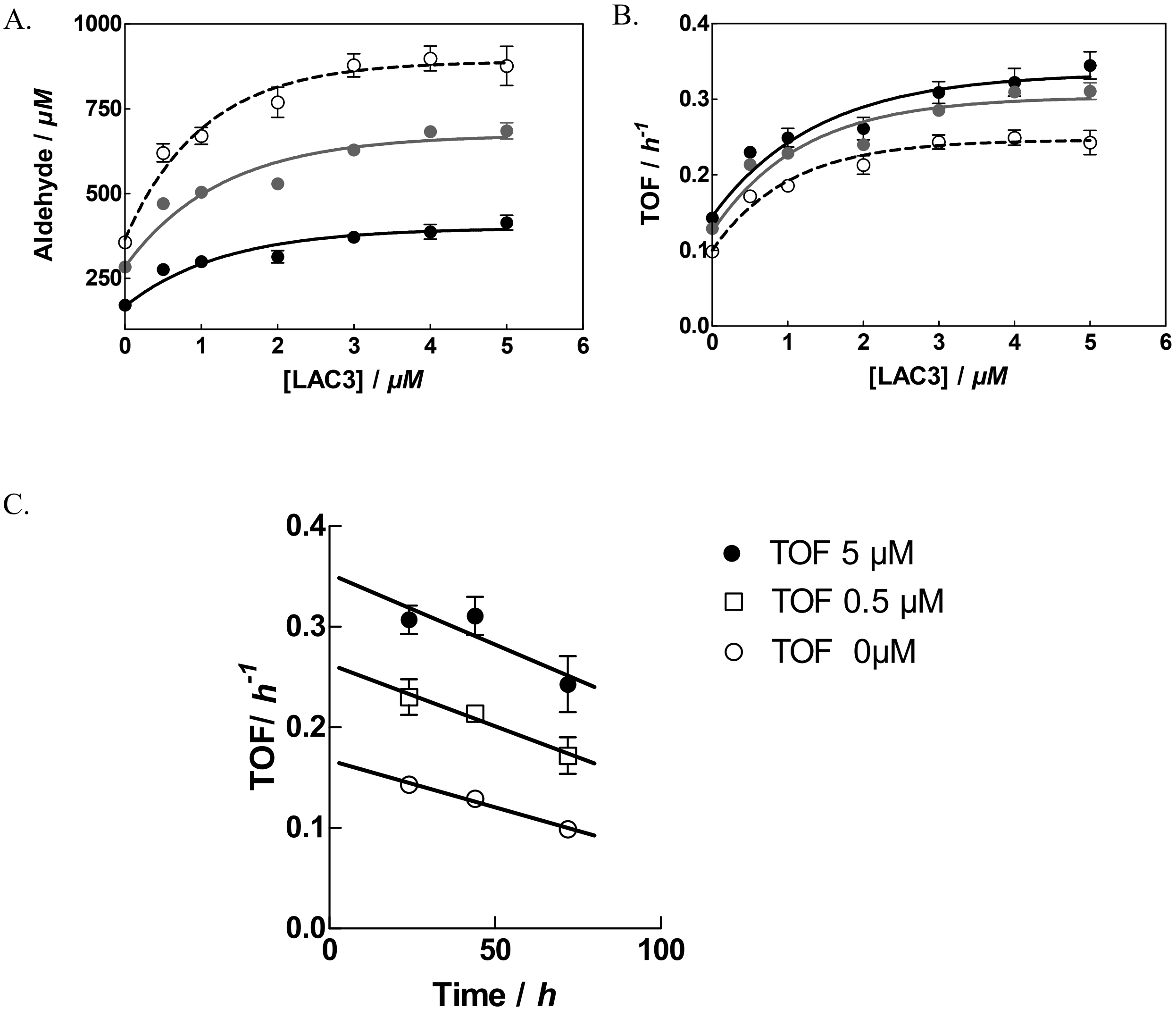

Figure SI6. Influence of reaction time on catalytic efficiencies at 25°C 50 µM complex **1** with 20 mM final substrate in the presence of 0 to 5 µM LAC3 in 33 mM acetate buffer pH 5.7 at 25 °C in 15 mL tubes with rotary shaking. A. Aldehyde production as a function of LAC3 concentration after 24 hours (—), 44 hours (—) and 72 hours (---). B. TOF (TOF= Turn over frequency in hr^{-1} = µmoles of product / (µmoles of complex 1 x time in hours)) as a function LAC3 concentration.C. TOF as function of time for 0 µM LAC3 (❍), 0.5 µM LAC3 (☐)and 5 µM LAC3 (•). Average of 3 sets of independent reactions.

Influence of different oxidation agents on alcohol oxidation

Quinones are well known re-oxidation agents for PD complexes. For the sake of comparison with LAC3, we checked the activity of the Pd complex (**1**) in the presence of an equimolar amount of benzoquinone ± 1 equivalent of $CuSO_4$. Results are presented Fig. SI7.

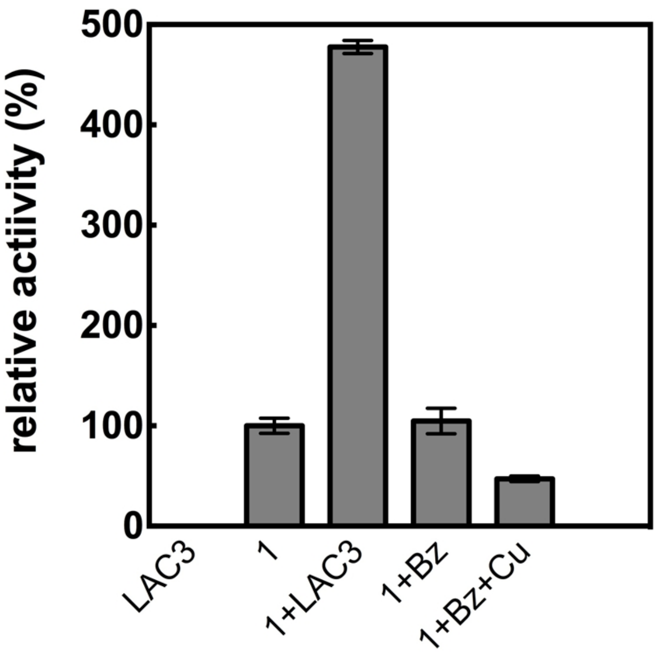

Figure SI7. Influence of different oxidation agents on alcohol oxidation. Reactions were performed in the presence of ±5 µM LAC3, ±50 µM complex **1**, ± 50 µM benzoquinone (Bz), ± $CuSO_4$ (Cu) and 100 mM final substrate concentration in 33 mM acetate buffer pH 5.7 at 25 °C in 15 mL tubes with rotary shaking for 24h. Average of 3 sets of independent reactions. The relative activity (%) is set to 100 for complex **1**.

Influence of catalyst load as function of time

The conversion of veratryl alcohol (20 mM veratryl alcohol at 22 °C in 33 mM acetate pH 5.7) into veratryl aldehyde was followed as function of time and catalyst load (from 0.125 to 2.5%). Values (average of three independent values) are presented in Table SI1.

Table SI1 : Conversion yield of veratryl alcohol

Load in **1** (%)	LAC3/**1**	24 hours	53 hours	125 hours
2.5	0	5.0 ± 0.1	6.9 ± 0.1	9.4 ± 0.2
	0.1	6.0 ± 0.3	8.8 ± 0.1	14.6 ± 0.2
1.25	0	2.9 ± 0.1	4.2 ± 0.0	6.5 ± 0.1
	0.1	4.5 ± 0.1	7.2 ± 0.3	10.6 ± 0.9
0.5	0	1.5 ± 0.0	2.2 ± 0.0	3.2 ± 0.1
	0.1	2.9 ± 0.1	4.8 ± 0.0	6.3 ± 0.2
0.25	0	0.9 ± 0.1	1.3 ± 0.1	2.0 ± 0.1
	0.1	1.9 ± 0.2	3.0 ± 0.3	4.2 ± 0.8
0.125	0	0.6 ± 0.0	0.7 ± 0.0	1.2 ± 0.1
	0.1	1.1 ± 0.0	1.8 ± 0.1	2.5 ± 0.1

[1] a) B.P. Buffin, J. P. Clarkson, N. L. Belitz, A. Kundu, J. mol. Catal A **2005**, *225*, 111-116; b) B.P. Buffin, N. L. Belitz, S. L. Verbeke, J.mol. Catal A **2008**, *225*, 149-154. ;

[2] ten Brink G.-J., Arends I. W. C. E., Sheldon R. A., Science **2000**, *287*, 1636-1639.

[3] (b) R. A. Sheldon, I. W. C. E. Arends, G.-J. ten brink and A. Dijksman Acc. Chem. Res., **2002**, *35*, 774-781; (c) G.-J. ten Brink, I. W. C. E. Arends, M. Hoogenraad,, G. Verspui, R. A. Sheldon, Adv. Synth. Catal. **2003**, *345*, 1341-1352.

[4] Y. Mekmouche, S. Zhou, A. M Cusano, E. Record, A. Lomascolo, V. Robert, A. J. Simaan, P. Rousselot-Pailley, F. Chaspoul and T. Tron, J. Biosc. Bioeng. **2014**, *117*, 25-27.

[5] U. K. Laemmli, *Nature*. **1970**, 227, 680
